# New Candidate for the Honor of the Discovery of the Application of Anæsthetic Inhalations to Surgery

**Published:** 1850-01

**Authors:** 


*New Candidate for the honor of the discovery of the application of anæsthetic inhalations to Surgery.*

The Southern Medical and Surgical Journal for December, 1849, contains a communication by Dr. C. W. Long, of Jefferson, Jackson county, Georgia, in which he claims priority in the application of the inhalation of the vapor of sulphuric ether to surgical operations. Dr. Long introduces affidavits setting forth that two tumours were removed by him from the neck, in a state of insensibility from the inhalation of sulphuric ether, as early as the year 1842. The affidavits are made by the patient, and by three persons who witnessed the operation. Since 1842, the writer states that he has employed the inhalations in one or more surgical cases annually.

To the inquiry which, as the writer remarks, will naturally arise in the mind of the reader, "why the results of the experiments in etherization were not sooner published," he replies as follows:

I was anxious, before making my publication, to try etherization in a sufficient number of cases to fully satisfy my mind that anæsthesia was produced by the ether, and was not the effect of the imagination, or owing to any peculiar insusceptibility to pain in the persons experimented on.

At the time I was experimenting with ether, there were physicians "high in authority," and of justly distinguished character, who were the advocates of mesmerism, and recommended the induction of the *mesmeric state* as adequate to prevent pain in surgical operations. Notwithstanding thus sanctioned, I was an unbeliever in the seience, and of the opinion that if the mesmeric state could be produced at all, it was only on "those of strong imagination and weak minds," and was to be ascribed solely to the workings of the patient's imaginations. Entertaining this opinion, I was the more particular in my experiments in etherization.

Surgical operations are not of frequent occurrence in a country practice, and especially in the practice of a young physician; yet I was fortunate enough to meet with two cases in which I could satisfactorily test the anæsthetic power of ether. From one of these patients, I removed three tumours the same day: the inhalation of ether was used only in the second operation, and was effectual in preventing pain, while the patient suffered severely from the extirpation of the other tumours. In the other case, I amputated two fingers of a negro boy: the boy was etherized during one amputation, and not during the other: he suffered from one operation, and was insensible during the other.

I have procured the certificates of the lady from whom the tumours were removed, and of her husband, who was present and witnessed the operations; and also that of the owner of the boy, establishing the fact of the insensibility of the patients to pain during these operations. These certificates were procured in preference to those establishing other operations, because they not only show that the experiments were continued from year to year, but also show that they were conducted so as to test the power of etherization.

After fully satisfying myself of the power of ether to produce anæsthesia, I was desirous of administering it in a severer surgical operation than any I had performed. In my practice, prior to the published account of the use of ether as an anæsthetic, I had no opportunity of experimenting with it in a capital operation, my cases being confined, with one exception, to the extirpation of small tumors, and the amputation of fingers and toes.

He adds:

While cautiously experimenting with ether, as cases occurred, with the view of fully testing its anæsthetic powers, and its applicability to severe, as well as minor, surgical operations, others, more favorably situated, engaged in similar experiments; and consequently the publication of etherization did not "bide my time." This being the case, I leave it with an enlightened medical profession to say, whether or not my claim to the dis-discovery of etherization is forfeited, by not being presented earlier, and with the decision which may be made, I shall be content.

The Editor of the Southern Journal states that "the writer of the communication is a highly worthy member of the medical profession, exceedingly modest in his pretensions, and entitled to full credit for all he advances."

As it is not to be presumed that the late Dr. Wells, of Hartford, Conn., or Drs. Jackson and Morton, of Boston, Mass., had any intimation of the experiments previously made by Dr. Long, whatever merit may belong to these gentlemen, severally or collectively, is in reality, in no wise diminished by the full recognition of the claim of Dr. Long to priority.

